# Iron status and inherited haemoglobin disorders modify the effects of micronutrient powders on linear growth and morbidity among young Lao children in a double-blind randomised trial

**DOI:** 10.1017/S0007114519001715

**Published:** 2019-03-08

**Authors:** Sonja Y. Hess, K. Ryan Wessells, Guy-Marino Hinnouho, Maxwell A. Barffour, Kanokwan Sanchaisuriya, Charles D. Arnold, Kenneth H. Brown, Charles P. Larson, Supan Fucharoen, and Sengchanh Kounnavong

**Affiliations:** 1 Program in International and Community Nutrition, Department of Nutrition, University of California, Davis, CA, USA; 2 Public Health Program, College of Health and Human Services, Missouri State University, Springfield, MO, USA; 3 Center for Research and Development of Medical Diagnostic Laboratories, Faculty of Associated Medical Sciences, Khon Kaen University, Khon Kaen, Thailand; 4 Canadian Coalition for Global Health Research, Ottawa, Canada; 5 Lao Tropical and Public Health Institute, Vientiane, Lao People’s Democratic Republic

**Keywords:** Micronutrient powder, Young children, Inherited Hb disorder, Iron status, Anaemia, Growth, Diarrhoea

## Abstract

Some studies found that providing micronutrient powder (MNP) causes adverse health outcomes, but modifying factors are unknown. We aimed to investigate whether Fe status and inherited Hb disorders (IHbD) modify the impact of MNP on growth and diarrhoea among young Lao children. In a double-blind controlled trial, 1704 children of age 6–23 months were randomised to daily MNP (with 6 mg Fe plus fourteen micronutrients) or placebo for about 36 weeks. IHbD, and baseline and final Hb, Fe status and anthropometrics were assessed. Caregivers provided weekly morbidity reports. At enrolment, 55·6 % were anaemic; only 39·3 % had no sign of clinically significant IHbD. MNP had no overall impact on growth and longitudinal diarrhoea prevalence. Baseline Hb modified the effect of MNP on length-for-age (LAZ) (*P* for interaction = 0·082). Among children who were initially non-anaemic, the final mean LAZ in the MNP group was slightly lower (–1·93 (95 % CI –1·88, –1·97)) *v*. placebo (–1·88 (95 % CI –1·83, –1·92)), and the opposite occurred among initially anaemic children (final mean LAZ –1·90 (95 % CI –1·86, –1·94) in MNP *v*. –1·92 (95 % CI –1·88, –1·96) in placebo). IHbD modified the effect on diarrhoea prevalence (*P* = 0·095). Among children with IHbD, the MNP group had higher diarrhoea prevalence (1·37 (95 % CI 1·17, 1·59) *v*. 1·21 (95 % CI 1·04, 1·41)), while it was lower among children without IHbD who received MNP (1·15 (95 % CI 0·95, 1·39) *v*. 1·37 (95 % CI 1·13, 1·64)). In conclusion, there was a small adverse effect of MNP on growth among non-anaemic children and on diarrhoea prevalence among children with IHbD.

Anaemia and deficiencies of Fe, iodine, Zn and vitamin A remain public health concerns in many low-income countries^(1)^. Multiple micronutrient powder (MNP) can be used for point-of-use fortification of foods consumed by young children and are recommended by the WHO to improve Fe status and reduce anaemia among children 6–23 months of age in populations where anaemia is a public health problem^(2)^. While the evidence for the positive impact of MNP on anaemia and Fe deficiency is consistent^(3,4)^, two recent meta-analyses of MNP reached different conclusions regarding morbidity outcomes. De Regil *et al.*^(3)^ found no effect of MNP on diarrhoea (risk ratio 0.97, 95 % CI 0.53, 1.78)), whereas Salam *et al.*^(4)^ found an increased risk in diarrhoea incidence with MNP (risk ratio 1.04, 95% CI 1.01, 1·06). Indeed there has been emerging evidence that an increased risk of infection, particularly from diarrhoea and malaria in areas of high transmission, may be associated with Fe supplementation^(5)^. For young children, WHO recommends MNP containing 10–12·5 mg of elemental Fe, 300 μg retinol and 5 mg of elemental Zn per sachet, with or without other micronutrients, and the provision of ninety sachets for consumption over a 6-month period^(2)^, though many programmes provide daily doses^(6)^. To ensure safety, the WHO recommends that MNP programmes also include behaviour change strategies to promote appropriate breastfeeding and complementary feeding practices, handwashing with soap, prompt attention to fever in malaria-endemic settings and measures to manage diarrhoea^(2)^. However, further research is needed to find safe ways of providing Fe-containing MNP.

Inherited Hb disorders (IHbD), consisting of structural Hb variants and thalassaemia, are found worldwide. The symptoms and clinical relevance of IHbD vary with the type and severity of the genetic defect and their effect on Hb structure and function. For example, *β*-thalassaemia is caused by partial or complete absence of *β*-globin synthesis due to mutations that affect transcription, translation and mRNA processing as well as gene deletion^(7)^. In Southeast Asia, severe Hb disorders include *α*^0^-thalassaemia, *β*^0^-thalassaemia, *β*-thalassaemia-Hb E disease and Hb H disease, all of which are associated with acute and chronic complications and serious health problems^(8,9)^. In addition, a large percentage of the population in Southeast Asia has non-severe IHbD, such as the *α*-thalassaemia trait (20–30%), the *β*-thalassaemia trait (3–9%) and the Hb E trait (up to 60 %)^(8,10)^. These latter milder forms of IHbD result from deletions or mutations in one or more, but not all, of the *α*- or *β*-globin alleles and often lead to only mild microcytosis, with or without anaemia^(11)^. A study of young Cambodian children found that homozygous Hb E was associated with an eighteen times higher risk of anaemia, and other heterozygous mutations were associated with a small but highly significant increased anaemia risk^(12)^. This latter study also found that heterozygous and homozygous Hb E, with or without *α*-thalassaemia trait, increased plasma ferritin and soluble transferrin receptor (sTfR) concentrations likely due to ineffective erythropoiesis and/or increased Fe absorption. Provision of supplementary Fe in foods may be contraindicated for some individuals with IHbD, as Fe absorption may not be normally regulated^(13–^^15)^. Thus, there is a risk that the insufficient down-regulation of Fe absorption in Fe-replete individuals with asymptomatic types of IHbD, which are prevalent in the populations of Southeast Asia, may cause adverse health outcomes when consuming supplementary Fe in products like MNP.

In view of these concerns about potential adverse effects of MNP on growth and morbidity in some populations^(4,5)^, and separate concerns raised about providing Fe to individuals with IHbD^(13,14)^, we tested a new MNP formulation, which contained a lower amount of Fe (6 mg/daily dose) and a higher amount of Zn (10 mg) than current formulations, along with thirteen other micronutrients. Main study outcomes are presented elsewhere^(16)^. Briefly, despite improving plasma Zn concentration, the provision of MNP had no impact on linear growth and the prevalence of diarrhoea and acute respiratory tract infections^(16,17)^. MNP improved the overall Fe status but tended to reduce anaemia only among children who were anaemic at baseline^(16)^.

The objectives of the present analyses were to (1) explore associations between IHbD and anaemia, micronutrient and growth status and morbidity burden; (2) explore whether the effects of a low-Fe-containing MNP on Fe status are modified by baseline Fe status and IHbD type; and (3) investigate whether baseline anaemia and Fe status and IHbD are potential risk factors for adverse effects of MNP on linear growth and morbidity.

We hypothesised that children with IHbD who receive MNP would have a smaller increase in Hb concentration and a greater increase in ferritin concentration than children without IHbD. Considering the reduced Fe content of the MNP, we further hypothesised that the product would be safe for all children participating in the study and that there would be no adverse impact of the intervention on growth and diarrhoea regardless of the baseline Fe status or presence of IHbD.

## Methods

### Study design and participants

The Lao Zinc study was designed as a randomised, double-blind controlled intervention trial. The present study was conducted according to the guidelines laid down in the Declaration of Helsinki, and all procedures involving human subjects were approved by the National Ethics Committee for Health Research, Ministry of Health, Lao People’s Democratic Republic (PDR; reference 039/NECHR), and the institutional review boards of the University of California, Davis, USA (reference 626187) and Khon Kaen University, Khon Kaen, Thailand (reference HE592006). Parental written informed consent was obtained from all subjects. The trial is registered as the Lao Zinc Study, NCT02428647 (https://clinicaltrials.gov). The trial was conducted from September 2015 to April 2017 in rural areas of Khammouane Province in Lao PDR.

The primary objective of the parent study was to determine the effects of two forms of daily preventive Zn supplementation *v*. therapeutic Zn supplementation for diarrhoea on young children’s physical growth and other health outcomes^(16)^.

The study procedures are described in detail elsewhere^(18)^. Briefly, written, informed consent was obtained by signature or fingerprint from one of the child’s primary caregivers (mother, father or legal guardian). In case the caregiver was illiterate, an impartial witness was present during the consent process, who confirmed that the information in the consent document was accurately explained to the participant and that the consent was freely given. Children were considered eligible if they were 6–23 months of age, their families accepted weekly home visits, planned to remain within the study area for the duration of the study and signed the informed consent document. Children were ineligible if they met one of the following criteria: severe anaemia (Hb < 70 g/l), weight-for-length z score (WLZ) < –3 SD^(19)^, presence of bipedal oedema, severe illness warranting hospital referral, congenital abnormalities potentially interfering with growth, chronic medical conditions (e.g. malignancy) requiring frequent medical attention, known HIV infection of the index child or the child’s mother, currently consuming Zn supplements or currently participating in any other clinical trial. A total of 3433 infants and young children 6–23 months of age were enrolled and individually randomised to one of four intervention groups using a computer-generated block randomisation scheme, with randomly selected block lengths of four or eight. The randomisation scheme was generated by a UC Davis statistician, and the study field workers assigned children following a list of study identification numbers. For the present analyses, only children randomised to the following two groups are considered: (1) the MNP group who received a daily preventive MNP containing 6 mg Fe, 10 mg Zn and thirteen other micronutrients or (2) the placebo control group who received daily placebo powder. The intervention products were distributed weekly for about 36 weeks. Both groups received low-osmolarity oral rehydration salt (ORS) solution for diarrhoea treatment. ORS was part of the diarrhoea treatment kit, which was given during enrolment, with instructions to store it in the home and use it for the treatment of a diarrhoea episode in the study child.

### Randomisation, masking and intervention products

The MNP and placebo powder sachets were produced by DSM Fortitech Asia Pacific (Banting, Malaysia). One MNP sachet provided the following micronutrients daily: 400 μg retinol activity equivalents (RAE) vitamin A, 0·5 mg thiamine, 0·5 mg riboflavin, 6 mg niacin, 0·5 mg vitamin B_6_, 150 μg dietary folate equivalents (DFE) folic acid, 0·9 μg cyanocobalamin, 30 mg ascorbic acid, 5 mg cholecalciferol, 5 mg tocopherol equivalents (TE) DL-*α*- tocopheryl acetate, 0·56 mg Cu as copper sulphate anhydrous, 90 μg iodine as potassium iodate, 6 mg Fe as ferrous fumarate, 17 μg Se as selenium selenite and 10 mg Zn as zinc gluconate. To ensure masking of the four intervention groups in the parent study^^(18)^^, all groups received a therapeutic tablet in case of diarrhoea, which were placebo tablets for the MNP and the placebo groups (produced by Nutriset SAS, Malaunay, France). To ensure blinding of investigators, study field staff and participating families, all intervention products were coded with a two-digit group code and a group-specific colour. Caregivers were instructed to add the entire content of the powder package into a single serving of semisolid or mashed food after the food had been cooked and cooled sufficiently to be eaten (but within 30 min of preparation). Caregivers were encouraged to mix the powder package into suitable foods such as mashed mango, banana and papaya, boiled pumpkin and boiled egg^(20)^.

### Procedures

To assess eligibility, children’s length, weight and mid-upper arm circumference (MUAC) were measured and Hb concentration was determined in a capillary blood sample (HemoCue^®^ Hb301; HemoCue AB). Maternal and household demographic and socio-economic characteristics of eligible children were recorded. Household food security was assessed using the Household Food Insecurity Access Scale^(21)^. Children’s anthropometric assessments were repeated after 36 weeks (end line). Unclothed or lightly dressed children were weighed to the nearest 20 g (SECA 383). Children’s recumbent length (SECA 416) and MUAC (left arm; Tri-Colored Single-Slotted Insertion Tape, Weigh and Measure) were measured to the nearest 0.1 cm. All measurements were collected in duplicate and the average of the two measurements was calculated. If measurements differed by >0.1 kg (weight), or >0.5 cm (length, MUAC), the measurement was repeated a third time and the average of the two closest measurements was calculated. Maternal weight (SECA 874) and height (SECA 213) were assessed once over the course of the study using a similar approach. Regular standardisation sessions were implemented to compare the performance of anthropometry teams among themselves and with their supervisors^(22)^; results of these standardisations have been reported previously^(16,23)^.

Children were visited weekly for delivery of intervention products and completion of a systematic, symptom-based, morbidity recall history, using a standardised data collection form. Specifically, caregivers reported stool number and consistency, among other symptoms during the preceding 7 d. Caregivers were instructed to initiate diarrhoea treatment (with ORS) whenever a child had >3 liquid or semi-liquid stools within a 24-h period. In addition, the morbidity surveillance worker reminded the caregiver to start or continue diarrhoea treatment as needed. If a child had persistent diarrhoea (>14 d), 20 mg Zn/d was provided for 10 d regardless of group assignment. Child feeding practices were assessed every 4 weeks with a brief FFQ to calculate selected WHO infant and young child feeding (IYCF) indicators, such as dietary diversity and minimum meal frequency^(24,25)^.

At baseline and end line, a venous blood sample was collected from children who were not acutely ill. The blood was first collected into an evacuated, trace element-free 7.5 ml polyethylene blood collection ement-free 7.5 ml polyethylene blood collection tube containing lithium heparin and, if adequate blood was available, into an evacuated 1.2 ml polyethylene blood collection tube containing EDTA (Sarstedt AG & Co.; reference 01.1604.400 and 06.1666.100, respectively). The blood samples were stored at 4–8°C and transported to the field project laboratory. The heparinised blood was centrifuged within 8 h of collection at 1097**g** (3100 rpm) for 10 min (PowerSpin Centrifuge Model LX C856; United Products & Instruments, Inc.). Plasma was aliquoted into 0.2 ml pre-labelled microcentrifuge tubes and stored at –20°C. Heparinised plasma samples were shipped on dry ice and remained frozen until analyses at the VitMin Lab (Willstaett, Germany) where ferritin, sTfR, C-reactive protein and α-1-acid glycoprotein concentrations were determined using a combined sandwich ELISA technique^(26)^.

The EDTA blood samples were collected at baseline for IHbD typing. These samples were transported to Thailand for detection of IHbD using an automated capillary zone electrophoresis (Capillarys II; Sebia) at Nakhon Phanom Hospital and PCR techniques at Khon Kaen University^(27,28)^. The types and levels of the following Hb fractions were determined: normal Hb (A, A2, F) and Hb variants (E and Constant Spring (CS)). Identification of *α*^0^-thalassaemia genes, including SEA and THAI deletions, was completed in all cases. Molecular screening for *β*-thalassaemia mutations was carried out in cases with either Hb A2 > 3.5 % or Hb EF phenotype. We determined the homozygosity of the Hb E phenotypes. In cases where no EDTA blood specimen was collected at baseline, we attempted to collect an EDTA blood sample at end line for IHbD typing.

### Definitions and expression of results

Anaemia and Fe deficiency were defined based on the following cut-offs: anaemia: Hb < 110 g/l; and Fe deficiency: ferritin < 12 µg/l and/or sTfR > 8.3 mg/l.

Length-for-age (LAZ), weight-for-age (WAZ), WLZ and MUAC (MUACZ) z scores were calculated according to the WHO 2006 growth standards^(19,29)^. Stunting, underweight and wasting were defined as < –2 sd LAZ, WAZ and WLZ, respectively. Diarrhoea was defined as the presence of ≥3 loose or liquid stools per 24 h.

Primary outcomes of the present analyses were the concentrations of Hb, ferritin and sTfR, length, weight, LAZ, WAZ and WLZ and diarrhoea prevalence; and secondary outcomes focused on the prevalence of anaemia, low ferritin, high sTfR, stunting, underweight and wasting. To investigate the modifying effects of IHbD on these outcomes, IHbD types were categorised based on Hb type and genetic results. All children with normal Hb type (A2A; Hb A_2_ ≤ 3·5 %, with negative PCR analysis for *α*^0^-thalassaemia) were classified as non-IHbD or non-clinically significant IHbD. Children with normal Hb type with positive results for an *α*^0^-thalassaemia gene mutation were categorised as *α*^0^-thalassaemia trait. The *β*-thalassaemia trait was diagnosed in cases with Hb A_2_ > 3·5 %. Homozygous Hb E was defined based on homozygosity testing. Thalassaemia disease was diagnosed in children with abnormal Hb profiles (A2ABart’sH, CSA2ABart’sH, EABart’s, CSEABart’s, EEBart’s, CSEEBart’s, EFBart’s, CSEFBart’s, EF, EFA) and/or as indicated by a complete analysis of *α*- and *β*-thalassaemia genes. To achieve adequate sample size, we combined the IHbD types into five categories (non-IHbD or non-clinically significant IHbD, *α*^0^-thalassaemia trait, *β*-thalassaemia trait or homozygous Hb E, Hb E trait and *α*- or HbE/*β*- or *αβ*-thalassaemia disease; Table 1) henceforth referred to as IHbD types. We further combined all IHbD into one category (the clinically significant IHbD, comprising *α*^0^-thalassaemia trait, Hb E trait, *β*-thalassaemia trait or homozygous Hb E or thalassaemia disease) and compared against all cases without clinically significant IHbD. Although the health consequences of Hb E trait are generally mild, this group was included in the ‘clinically significant IHbD’ group due to its high prevalence in the study population and thus the potential relevance for public health programmes, considering the distribution of MNP.

**Table 1:** Definitions of inherited Hb disorders (IHbD)

Definition based on Hb type and genetic results	IHbD type	Presence/absence of IHbD
A2A or CSA2A, Hb A_2_ ≤ 3.5% Negative *α*^0^-thalassaemia gene	Non-IHbD or non-clinically significant IHbD	Non-IHbD or non-clinically significant IHbD
A2A, Hb A_2_ ≤ 3.5% Positive *α*^0^-thalassaemia gene	*α*^0^-Thalassaemia trait	Clinically significant IHbD
EA, Hb E < Hb A Positive *α*^0^-thalassaemia gene	*α*^0^-Thalassaemia trait	Clinically significant IHbD
EA or CSEA, Hb E < Hb A Negative *α*^0^-thalassaemia gene	Hb E trait	Clinically significant IHbD
A2A, Hb A_2_ > 3.5 % Negative *α*0-thalassaemia gene	*β*-Thalassaemia trait or homozygous Hb E	Clinically significant IHbD
EE or CSEE, EF or CSEF (with positive testing for Hb E homozygote) Either negative or positive *α*0-thalassaemia gene	*β*-Thalassaemia trait or homozygous Hb E	Clinically significant IHbD
*α*-Thalassaemia disease: A2ABart’sH or CSA2ABart’sH Hb E/*β*-thalassaemia disease: EF, EFA (with Hb E > Hb A) *αβ*-Thalassaemia disease: EABart’s or CSEABart’s EEBart’s or CSEEBart’s EFBart’s or CSEFBart’s All cases were confirmed by DNA analysis	Thalassaemia disease	Clinically significant IHbD

CS, Constant Spring.

### Sample size

Because objectives 2 and 3 of the present analyses were to examine the potential modifying effects of Fe status and IHbD on response to MNP, the sample size for the present analyses was based on the full sample size available for the MNP and the placebo groups in the parent study. As described previously^(18)^, a total of 710 children were needed in each of the treatment groups. Allowing for 15 % attrition, we needed to enrol 835 children per group, which we rounded up to 850 per group. For the present two-group analyses, this would allow the detection of an effect size of 0.18 (*α* = 0.05, *β* = 0.10) for the main outcomes (anaemia, Fe status, growth and morbidity), and the detection of a difference in effect size of about 0.35 (*α* = 0.05, *β* = 0.10) for the modifying effect of baseline Fe status or IHbD on the main effects.

### Statistical analyses

A statistical analysis plan was posted online prior to commencing data analyses^(30)^. Primary analyses were performed using R version 3.4. Treatment effects were assessed in minimally adjusted models including baseline measurement (if collected), age of the child at enrolment and district of enrolment. Treatment effects were assessed at the 5 % level of significance. In secondary analyses, we created adjusted models that included covariates strongly associated with the outcome to potentially improve the precision of our estimates (i.e. decrease standard errors). We considered a list of pre-specified covariates for inclusion if they were associated with the outcome at a 10 % significance level in bivariate analyses.

ANCOVA models were used to assess continuous anthropometry outcomes, Hb concentration and log transformed Fe status indicators, while modified Poisson regression was used to assess all dichotomous outcomes. Negative binomial models with an offset were used for the morbidity outcomes.

A pre-specified list of effect modifiers was assessed by including an interaction term in the minimally adjusted models. Marginally significant interactions (*P* < 0.1) were further examined with stratified analyses, estimation of separate regression lines or estimation of adjusted means at certain values of the effect modifier, in order to understand the nature of the effect modification. For illustrative purposes, we present figures of the stratified analyses for outcomes with significant or marginally significant effect modifications.

Baseline measures were compared across IHbD groups by testing the global null hypothesis of any differences by group using a likelihood ratio test, controlling for child’s age at enrolment and district of residence. Group estimates were then qualitatively assessed to describe clinically meaningful patterns across groups.

Both Fe status indicators (plasma ferritin, sTfR) were adjusted for inflammation by adapting procedures recommended by the Biomarkers Reflecting Inflammation and Nutritional Determinants of Anemia (BRINDA) group^(31,32)^. Specifically, adjustment factors reflecting the changes in nutritional biomarkers during inflammation were determined by pooling together the samples collected at baseline from all four intervention groups of the Lao Zinc Study^(16)^. The adjustment factors (i.e. regression coefficient for C-reactive protein and/or *α*-1-acid glycoprotein) were subsequently applied to both baseline and end line samples. In adapting the BRINDA approach, we used the 10th percentile of the C-reactive protein and a-1-acid glycoprotein concentrations in this population as a cut-off for determining whether adjustment was necessary. Only inflammation-adjusted Fe status results will be presented here.

## Results

### Baseline characteristics of study population

**Fig. 1:**
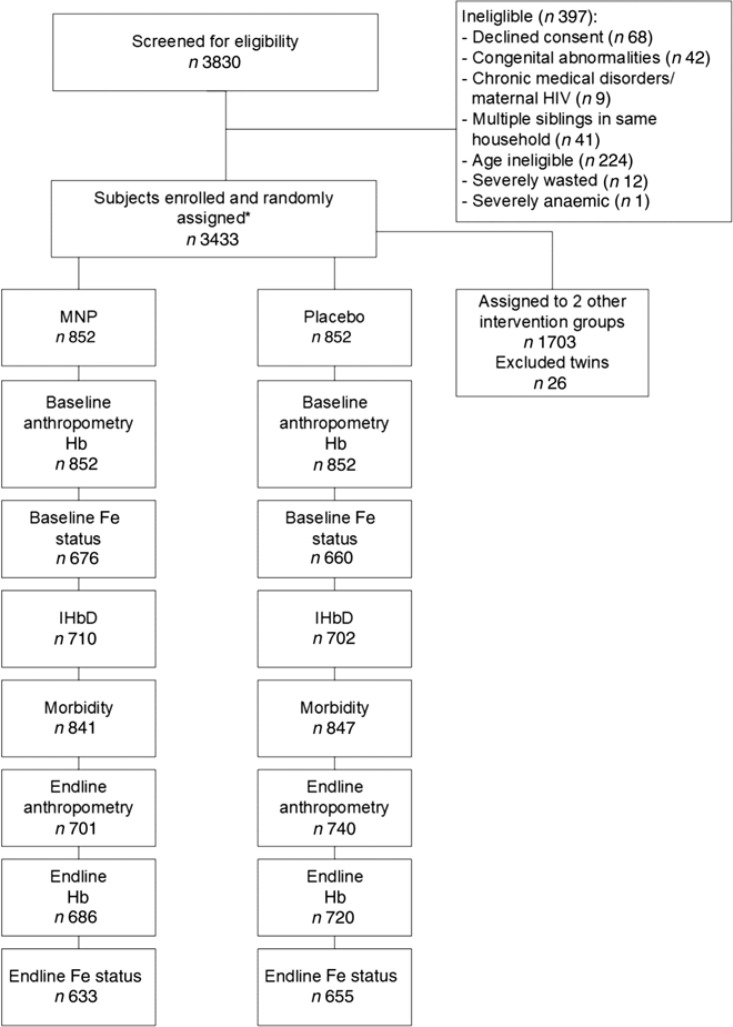
Flow chart of study participants. * The main trial included four intervention groups; only two of these are included in the present analysis, for which details are shown here. MNP, micronutrient powder; IHbD, inherited Hb disorders.

A total of 3830 children were screened for eligibility for the parent study, and 3433 were enrolled and assigned to one of the four intervention groups, of which 852 were assigned to the MNP and placebo control groups. The final sample size included in the present analyses depended on the specific outcome (Fig. 1). Attrition was similar across intervention groups, with the largest difference between groups occurring for end line anthropometry where MNP had 82.3 % and placebo had 86.9 % follow-up, which resulted in a difference of 4.6 %. Reasons for loss appear to be similar across intervention arms. The most common recorded reasons were ‘withdrawal with no reason provided’ making up 52.9% and ‘moving’ making up 27.0%. MNP had a higher rate of withdrawal due to vomiting (*n* 19, 12.6% of MNP withdrawals compared with *n* 2,1 .8 % of placebo withdrawals). When comparing the baseline characteristics of children with end line anthropometric outcomes, there were no significant differences among the two groups (Supplementary Table S1). Among children enrolled in the two groups (*n* 1704), 1336 provided a venous blood sample at baseline for assessment of Fe status, and IHbD was assessed in 1412 children.

The mean age of children at enrolment was 14.2 (sd 5.0) months and the mean age of their mothers was 26.9 (sd 6.0) years (Table 2). Reported IYCF practices at baseline indicated that only 15.3% had adequate dietary diversity, but more than half reported achieving minimum meal frequency and consumption of Fe-rich foods (59.1 and 66.7 %, respectively). These indicators of IYCF improved somewhat over the course of the study, but adequate dietary diversity was still only reported on about a third of observation days.

**Table 2:** Baseline characteristics of young children, mothers and households (Mean values and standard deviations; numbers of participants; percentages)

	All			MNP			Placebo		
	Mean		SD	Mean		SD	Mean		SD
*n*		1704			852			852	
Children									
Enrolment age (months)	14.2		5.0	14.2		5.0	14.1		5.1
Male (%)		50.4			49.5			51.3	
Adequate dietary diversity at baseline^*^(%)		15.3			14.1			16.5	
Minimum meal frequency at baseline^*^(%)		59.1			60.3			58.0	
Consumption of Fe-rich foods at baseline^*^(%)		66.7			66.9			66.5	
Proportion of days with adequate dietary diversity^†^	0.30		0.28	0.30		0.28	0.29		0.28
Proportion of days with minimum meal frequency^†^	0.62		0.36	0.61		0.36	0.62		0.35
Proportion of days with Fe-rich foods^†^^‡^	0.85		0.20	0.85		0.19	0.84		0.20
Mothers									
Age (years)	26.9		6.0	26.9		5.9	27.0		6.2
Maternal height (cm)	150.6		5.2	150.5		5.1	150.7		5.3
Maternal BMI (kg/m2)	21.4		2.9	21.4		2.9	21.4		3.0
Years of education	4.6		3.5	4.7		3.5	4.6		3.5
Buddhist (%)		89.1			88.2			90.1	
Married (%)		95.7			96.4			94.9	
Households									
Food secure^§^(%)		34.2			34.4			34.0	
Number of children under 5 years	1.5		0.7	1.4		0.6	1.5		0.7
Improved walls and floor (%)		31.9			32.0			31.8	
Number of rooms	2.5		1.0	2.5		1.0	2.5		1.0
Number of poultry owned	9.3		11.1	9.2		10.9	9.5		11.2

MNP, micronutrient powder; IYCF, infant and young child feeding.

* Baseline IYCF practices during 24 h prior to enrolment^(24,25)^.

† IYCF practices assessed every 4 weeks throughout the study; proportion of observed days with the positive feeding behaviour during study.

‡ Fe-rich foods did not include the MNP provided by the study.

§ Food security assessed using the Household Food Insecurity Access Scale^(21)^.

Approximately one-third of the children (39.3 %) had no evidence of a clinically significant IHbD, 2.8 % had *α*^0^-thalassaemia trait, 42.7 % had Hb E trait, 13.4 % had *β*-thalassaemia trait or were homozygous for Hb E and 1 .8 % had *α*- or *β*-thalassaemia disease (Table 1). Overall anaemia prevalence was 55.6 % at baseline. All children with thalassaemia disease were anaemic, and three fourths of the children with *α*^0^-thalassaemia trait, *β*-thalassaemia trait and homozygous Hb E were anaemic compared with an anaemia prevalence of 46.8 % among children without clinically significant IHbD (*P* < 0.001; Table 3). Ferritin marginally differed by IHbD group with higher concentrations among children with any type of IHbD compared with those without. High sTfR status was more common among children with *α*^0^-thalassaemia trait, *β*-thalassaemia trait or homozygous Hb E and *α*- or *β*-thalassaemia disease (63.6 to 100%) than among those without IHbD (53.3–56.1 %). LAZ at baseline and prevalence of underweight also significantly differed by IHbD group, with *α*- or *β*-thalassaemia diseased children having lower LAZ (–2.1 v. roughly –1.7) and higher prevalence of underweight (42.3 v. roughly 25 %; Table 3).

**Table 3:** Baseline Hb concentration, anaemia prevalence, iron status concentrations and anthropometries by type of inherited Hb disorders (IHbD) among young children participating in the Lao Zinc Study (Mean values and standard deviations; numbers and percentages; medians and quartiles 1 and 3)

	Non-clinically significant IHbD			*α*^0^-Thalassaemia trait			Hb E trait		*β*-Thalassaemia trait or homozygous Hb E			*α*- or *β*- Thalassaemia disease			P^†^
	Mean		SD	Mean		SD	Mean	SD	Mean		SD	Mean		SD	
Baseline Hb (*n*)		554			40			603		189			26		
Hb (g/l)	109.9		10.4	105.9		80	107.8	100	1030		9.4	93.7		9.3	<0.001^*^
Anaemia (Hb < 110 g/l)															<0.001^*^
*n*		259			30			331		140			26		
%		46.8			75.0			54.9		74.1			1000		
Baseline Fe status‡ (*n*)		473			33			515		154			23		
Ferritin (µg/l)															0.090
Median		16.6			18.4			18.6		220			21.1		
Q1, Q3		8.6, 30.1			12.2, 27.7			9.5, 33.1		11.1, 34.1			12.0, 37.0		
Low ferritin (pF < 12 µg/l)															0.338
*n*		166			8			169		44			6		
%		35.1			24.2			32.8		28.6			26.1		
sTfR (mg/l)															<0.001^*^
Median		8.54			9.38			8.64		9.84			17.31		
Q1, Q3		7.14, 10.80			7.56, 10.58			7.28, 10.75		8.56, 12.40			13.34, 25.43		
High sTfR (sTfR > 8.3 mg/l)															<0.001^*^
*n*		252			21			289		122			23		
%		53.3			63.6			56.1		79.2			1000		
Baseline anthropometry (*n*)		554			40			603		189			26		
Length (cm)	73.0		5.42	72.3		5.18	72.7	5.42	72.8		5.47	71 .5		5.50	0.039^*^
LAZ	–1 .58		1 .06	.1 .63		0.96	–1 .74	1 .05	–1.81		1 .02	–2.08		1 .00	0.016^*^
Stunting (LAZ < –2 sd)															0.087
*n*		194			11			236		79			14		
%		350			27.5			39.1		41.8			53.8		
Weight (kg)	8.38		1 .28	8.36		1.18	8.35	1 .29	8.36		1 .23	8.21		1 .64	0.712
WAZ	–1.36		0.93	–1 .26		0.96	–1 .40	0.98	–1 .46		0.95	–1 .49		1 .23	0.635
Underweight (WAZ < –2 SD)															0.016^*^
*n*		123			10			161		47			11		
%		22.2			250			26.7		24.9			42.3		
WLZ	.0.74		0.90	–0.55		0.87	–0.68	0.93	–0.71		0.89	–0.55		1 .26	0.606
Wasting (WLZ < –2 SD)															0.826
*n*		39			2			50		12			2		
%		7.0			5.0			8.3		6.3			7.7		
MUAC (cm)	13.8		0.95	13.9		100	13.8	0.99	13.8		0.92	13.8		1.26	0.884
MUACZ	–0.65		0.84	–0.55		0.89	–0.68	0.87	–0.72		0.83	–0.62		1 .08	0.894
Low MUACZ (MUACZ < –2 SD)															0.331
*n*		26			1			40		14			3		
%		4.7			2.5			6.6		7.4			11.5		

pF, plasma ferritin; sTfR, soluble transferrin receptor; LAZ, length-for-age z score; WAZ, weight-for-age z score; WLZ, weight-for-length z score; MUAC, mid-upper arm circumference; MUACZ, mid-upper arm circumference z score.

* Statistically significant (*P* < 0·05).

† P value for bivariate association controlling for child age at enrolment and district.

‡ Fe status indicators adjusted for inflammation.

### Effects of micronutrient powder on children’s Hb and iron status and effect modification by baseline Hb and iron status and inherited Hb disorders

Overall, MNP significantly increased Hb concentration compared with placebo^(16)^ (Supplementary Table S2). However, this resulted in only a marginally lower final anaemia prevalence among children who received MNP (39.7 %) compared with children in the placebo group (43.2 %; *P* = 0.067). Children in the MNP group had a significantly greater increase in ferritin and a reduction in sTfR concentrations compared with those in the placebo group. At end line, 20.3 % children in the MNP group had low ferritin compared with 30.7 % in the placebo group (*P* < 0.001). Similarly, the prevalence of elevated sTfR was lower in the MNP group compared with the placebo group (45.2 *v.* 53.3 %; *P* = 0.006).

**Fig. 2:**
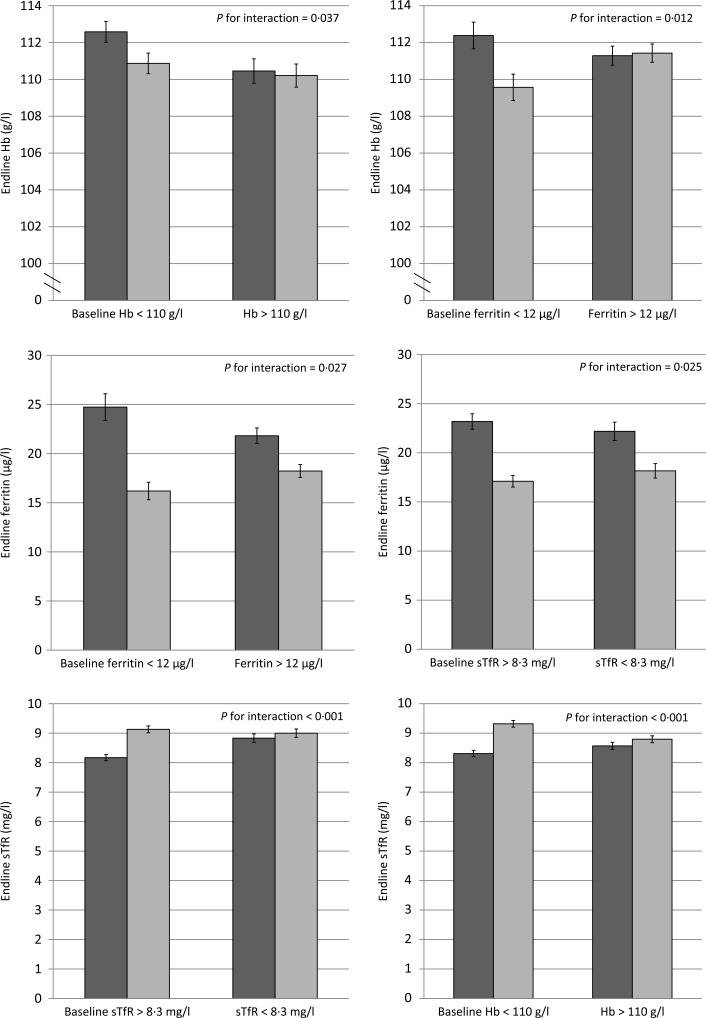
Effect modification of baseline anaemia and iron status on Hb and iron status at end line among young children who received either a low-iron-, high-zinc-containing micronutrient powder (▪) or a placebo powder (◻). Only selected significant or marginally significant effect modifications are shown; a complete overview of effect modifications is included in Table 4. Iron status indicators adjusted for inflammation. Continuous outcomes log transformed for analysis. sTfR, soluble transferrin receptor. Values are means, with standard deviations represented by vertical bars.

We tested for effect modification of baseline Hb and Fe status and IHbD on the impact of MNP on Hb, anaemia, Fe status and Fe deficiency (Table 4). The impact of MNP on end line Hb was significantly modified by baseline Hb and ferritin concentrations (*P* for interaction = 0·037 and 0·012, respectively; Fig. 2). Specifically, children who were anaemic at baseline had a greater Hb response to MNP than placebo, but there was no difference in the Hb response among non-anaemic children. Likewise the effects of MNP on ferritin and sTfR concentrations at end line were both significantly greater among children who were Fe deficient at baseline.

**Table 4:** Significance of effect modification of responses to micronutrient powder (MNP) by baseline Hb and iron status, inherited Hb disorder (IHbD) type and the presence and absence of IHbD on final study outcomes among young children who received either a low-iron, high-zinc MNP or a placebo powder in the Lao Zinc Study

	Baseline Hb	Baseline ferritin^†^	Baseline sTfR^†^	IHbD type^‡^	Presence/absence of IHbD^§^
Hb	0·037^*^	0·012^*^	0·152	0·557	0·146
Anaemia	0·935	0·014^*^	0·401	—	0·409
Ferritin^†^	0·113	0·027^*^	0·025^*^	0·103	0·255
Low ferritin^†^	0·158	0·166	0·941	—	0·062^*^
sTfR^†^	<0·001^*^	<0·001^*^	<0·001^*^	0·909	0·645
High sTfR^†^	0·690	0·134	0·032^**^	—	0·062^*^
Length	0·141	0·604	0·117	0·813	0·849
LAZ	0·082^*^	0·624	0·307	0·789	0·802
Stunting	0·323	0·677	0·882	0·355	0·786
Weight	0·508	0·238	0·207	0·496	0·150
WAZ	0·425	0·407	0·336	0·664	0·202
Underweight	0·639	0·644	0·629	0·601	0·970
Weight-for-length	0·582	0·520	0·274	0·716	0·353
WLZ	0·983	0·285	0·746	0·676	0·208
Wasting	0·596	0·668	0·615	—	0·019^**^
MUAC	0·038^**^	0·252	0·553	0·175	0·659
MUACZ	0·037^**^	0·280	0·585	0·225	0·693
Low MUACZ	0·164	0·878	0·215	—	0·321
Diarrhoea longitudinalprevalence	0·508	0·305	0·352	0·247	0·095^*^
Diarrhoea incidence	0·178	0·495	0·669	0·292	0·274

sTfR, soluble transferrin receptor; –, not available because model did not converge; LAZ, length-for-age z score; WAZ, weight-for-age z score; WLZ, weight-for-length z score; MUAC, mid-upper arm circumference; MUACZ, MUAC z score.

Statistical significance: * *P* < 0·10, ** *P* < 0·05.

† Fe status indicators adjusted for inflammation. Continuous outcomes log transformed for analysis.

‡ IHbD types included five categories: *α*^0^-thalassaemia trait, *β*-thalassaemia or homozygous Hb E, Hb E trait, thalassaemia disease, non-clinically significant IHbD.

§ All IHbD were combined into one category (i.e. *α*- or *β*-thalassaemia or Hb E) and compared against all cases without clinically significant IHbD.

**Fig. 3:**
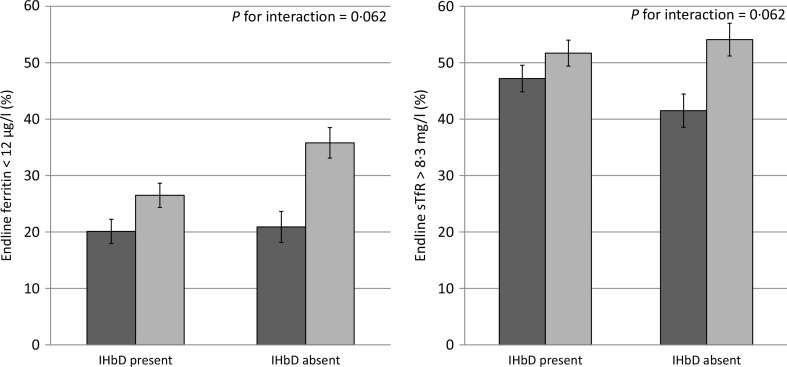
Effect modification of inherited Hb disorder (IHbD) on final iron status among young children who received either a low-iron, high-zinc micronutrient powder (◻) or a placebo powder (◻). Only significant or marginally significant effect modifications are shown; a complete overview of effect modifications is included in Table 4. Iron status indicators adjusted for inflammation. Continuous outcomes log transformed for analysis. All IHbD were combined into one category and compared against all cases without clinically significant IHbD. sTfR, soluble transferrin receptor. Values are means, with standard deviations represented by vertical bars.

As mentioned above, the modifying effect of IHbD was explored by considering both the five IHbD types and the presence/absence of clinically significant IHbD. Neither the IHbD type nor the presence/absence of IHbD modified the impact of MNP on Hb, ferritin and sTfR concentrations at end line (Fig. 3). In addition, the presence/absence of IHbD did not modify the impact of MNP on the prevalence of anaemia at end line. However, there was a marginally significant modifying effect of the presence/absence of IHbD on the effect of MNP on the prevalence of low ferritin and high sTfR concentrations at end line (*P* for interaction = 0.062 and 0.062, respectively). In particular, among children in the placebo group, those with clinically significant IHbD had a lower final prevalence of Fe deficiency (based on low ferritin) than those without clinically significant IHbD (26.5 (95% CI 22.2, 30.9)%) *v*. 35.8 (95% CI 30.4, 41.3)%). In the MNP group, there was a greater response to MNP among those without IHbD. This resulted in a final prevalence of Fe deficiency (based on low ferritin), which was the same in both sets of children who received MNP, regardless of their IHbD status (20.9 (95% CI 15.4, 26.4)% *v*. 20.1 (95% CI 15.8, 24.5) %). A similar pattern was seen with regard to the final prevalence of elevated sTfR. The impact of MNP on the prevalence of high sTfR was largest among children without clinically significant IHbD (41.5 (95 % CI 35.6, 47.3) %), compared with children with IHbD (47.2 (95 % CI 42.5, 51.8) %). In comparison, children in the placebo group had a slightly higher prevalence of elevated sTfR (54.1 (95 % CI 48.3, 59.9) %) among those without clinically significant IHbD *v.* those with IHbD (51.7 (95% CI 47.1, 56.3)%). Because the models did not converge to assess the modifying effect of IHbD type on the final prevalence of anaemia, low ferritin and high sTfR (Table 4), we investigated the impact of MNP on these prevalence in stratified analyses by those IHbD types with adequately large sample sizes (non-clinically significant IHbD, *n* 475; Hb E trait, *n* 530; *β*-thalassaemia trait or homozygous Hb E, *n* 167). The impact of MNP on anaemia prevalence at end line was not modified by the IHbD type, and the prevalence of Fe deficiency (low ferritin and high STfR) was significantly reduced only in children without clinically significant IHbD (Table 5). We were unable to draw conclusions regarding the modifying effects of *α*^0^-thalassaemia trait and *α*- or *β*-thalassaemia disease on the prevalence of anaemia and Fe deficiency due to the small sample size of these IHbD subgroups.

**Table 5:** Effect of a low-iron-, high-zinc-containing micronutrient powder (MNP) on the prevalence of anaemia, iron deficiency and wasting by inherited Hb disorder (IHbD) type† (Numbers of participants, percentages, prevalence ratios for comparison values and 95 % confidence intervals)

		MNPs					Placebo				Minimally adjustedcomparison	95 % CI‡
		Baseline			End line		Baseline		End line			
		*N*	*n*	%	*n*	%	*n*	%	*n*	%		
Anaemia (%) (Hb < 110 g/l)	Non-clinically significant IHbD	475	111	48.3	68	29.6	110	44.9	85	34.7	0.79	0.62, 1.01
	Hb E trait	530	158	60.3	101	38.5	134	50.0	108	40.3	0.89	0.73, 1.10
	*β*-Thalassaemia trait or homozygous Hb E	167	60	72.3	52	62.7	63	75.0	57	67.9	0.96	0.77, 1.18
Low ferritin (%) (pF < 12 μg/l)§	Non-clinically significant IHbD	374	68	37.0	42	22.8	70	36.8	72	37.9	0.56^*^	0.42, 0.75
	Hb E trait	420	74	35.9	42	20.4	66	30.8	52	24.3	0.83	0.60, 1.16
	*β*-Thalassaemia trait or homozygous Hb E	128	19	26.8	8	11.3	19	33.3	15	26.3	0.60	0.28, 1.29
High sTfR (%) (sTfR > 8.3 mg/l)§	Non-clinically significant IHbD	374	100	54.3	72	39.1	100	52.6	94	49.5	0.75^*^	0.61, 0.92
	Hb E trait	420	123	59.7	89	43.2	114	53.3	95	44.4	0.88	0.73, 1.06
	*β*-Thalassaemia trait or homozygous Hb E	128	56	78.9	43	60.6	48	84.2	45	78.9	0.85	0.68, 1.06
Wasting (%) (WLZ < –2 SD)	Non-clinically significant IHbD	497	17	7.2	15	6.4	18	7.2	9	3.6	2.87^*^	1.21, 6.83
	Hb E trait	537	25	9.4	15	5.7	21	7.7	14	5.1	0.86	0.46, 1.63
	*β*-Thalassaemia trait or homozygous Hb E	172	10	11.9	5	6.0	2	2.4	2	2.4	0.70	0.16, 3.11

pF, plasma ferritin; sTfR, soluble transferrin receptor; WLZ, weight-for-length z-score.

* Statistically significant difference between the MNP and the placebo group (*P* < 0·05).

† Because the model to explore effect modification did not converge for the prevalence of anaemia, low ferritin and high sTfR and wasting, we investigated the impact of MNP on these prevalence (and the concentrations of the respective indicators) in stratified analyses by those IHbD types, for which we had reasonably large sample sizes.

‡ Adjusted for baseline value, age at enrolment, and regional district.

§ Fe status indicators adjusted for inflammation. Continuous outcomes log transformed for analysis.

### Effects of micronutrient powder on children’s growth and effect modification by baseline iron status and inherited Hb disorders

**Fig. 4:**
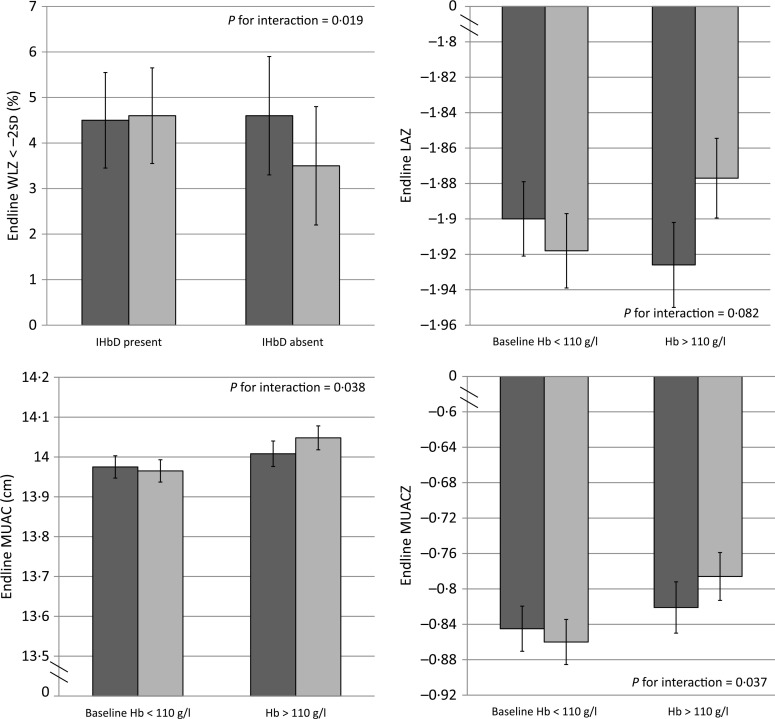
Effect modification of inherited Hb disorder (IHbD) and baseline anaemia on selected growth outcomes among young children who received either a low-iron-, high-zinc-containing micronutrient powder (◻) or a placebo powder (◻). Only selected significant or marginally significant effect modifications are shown; a complete overview of effect modifications is included in Table 4. All IHbD were combined into one category (i.e. *α*- or *β*-thalassaemia or Hb E) and compared against all cases without clinically significant IHbD. WLZ, weight-for-length z score; LAZ, length-for-age z score; MUAC, mid-upper arm circumference; MUACZ, mid-upper arm circumference z score. Values are means, with standard deviations represented by vertical bars.

Overall, MNP had no impact on any of the growth outcomes at end line compared with placebo^(16)^ (Supplementary Table S3). The impact of MNP on some growth outcomes (LAZ, MUAC, MUACZ) was modified by baseline Hb (Fig. 4), but not Fe status (Table 4). There was an adverse effect of MNP on some growth outcomes among non-anaemic children, as the final mean LAZ in the MNP group was slightly lower (–1·93 (95 % CI –1·88, –1·97)) *v.* the placebo group (–1·88 (95 % CI–1·83, –1·92); *P* for interaction = 0·082); similarly, the final mean MUAC was slightly smaller in the MNP group (14·01 (95 % CI 13·94, 14·07) cm) *v.* the placebo group (14·05 (95 % CI 13·99, 14·11) cm; *P* for interaction = 0·038). On the other hand, among the initially anaemic children, there was a tendency of a potential beneficial impact, though the magnitude was very small on the final mean LAZ (–1·90 (95 % CI –1·86, –1·94) in MNP *v.* –1·92 (95 % CI –1·88, –1·96) in placebo) and the final mean MUAC (13·98 (95 % CI 13·92, 14·03) cm in MNP *v.* 13·96 (95 % CI 13·91, 14·02) cm in placebo). The presence/absence of IHbD did not modify the effect of MNP on growth, except for wasting (*P* for interaction = 0·019; Fig. 4). Among children with IHbD, there was no effect of MNP on wasting. However, among children without clinically significant IHbD, those who received MNP had a higher final prevalence of wasting (6·4 %) than those who received placebo (3·6 %).

### Effects of micronutrient powder on children’s diarrhoeal morbidity and effect modification by baseline iron status and inherited Hb disorders

There was no overall difference in longitudinal diarrhoea prevalence and diarrhoea incidence between treatment groups (Table 6). However, IHbD status modified the intervention effect on diarrhoea prevalence marginally (*P* = 0·095; Table 4). Among children with s IHbD, those in the MNP group had higher diarrhoea prevalence compared with those in the placebo group (1.37 (95 % CI 1.17,1.59) *v.* 1.21 (95 % CI 1.04,1.41)); by contrast among children without IHbD those who received MNP had a lower diarrhoea prevalence compared with those in the placebo group (1.15 (95% CI 0.95, 1.39) *v.* 1.37 (95% CI 1.13, 1.64)).

**Table 6:** Effect of a low-iron, high-zinc micronutrient powder (MNP) on diarrhoea incidence and prevalence of young children in the Lao Zinc Study (Medians, quartiles 1 and 3; numbers of participants; mean values and standard deviations; rate ratios for comparison and 95 % confidence intervals)

	MNP		Placebo					
	Median	Q1, Q3	Median	Q1, Q3	Minimally adjusted^*^ comparison	95 % CI	Fully adjustedt comparison†	95 % CI
*n*	841		847					
Days observed per child	252	238,	252	245,				
Diarrhoea days per child	1	252		252				
Longitudinal diarrhoea prevalence per child‡		0, 3	1	0, 4	1.00	0.86, 1.16	0.96	0.84, 1.14
Mean	1.50		1.38					
SD	3.97		2.70					
Days at risk per child	246	231,	247	236,				
		252		1.00				
Diarrhoea episodes per child	1	0, 2	1	0, 2				
Diarrhoea incidence ratio per child^§^	0.86	3.07	0.70	1.25	1.00	0.88, 1.13	1.00	0.88, 114

* Minimally adjusted for baseline value, age at enrolment and regional district.

† Fully adjusted includes adjustments for minimal adjustment variables and potentially for maternal age, height, BMI, and education, child sex, month of enrolment, adequate dietary diversity at baseline, minimum meal frequency at baseline, Fe-rich foods consumed at baseline, number of children under 5 years in the household, household food insecurity score, socio-economic index, average adequate dietary diversity, average consumption of Fe-rich foods, and baseline growth status.

‡ Days with diarrhoea in 100 d observed. Diarrhoea defined as 24 h with at least three loose or liquid stools.

§ Episodes of diarrhoea in 100 d at risk of having a diarrhoea episode defined as a period of diarrhoea followed by 2 d diarrhoea free.

## Discussion

Among young children in the central rural Lao PDR, the anaemia prevalence and the presence of IHbD were high. Daily provision of a low-Fe-, high-Zn-containing MNP to young Laotian children resulted in significantly increased Hb and ferritin and decreased sTfR concentrations after 9 months of supplementation. The impact on Hb and Fe status indicators was significantly greater among those with lower Hb and lower Fe status at baseline and those without clinically significant IHbD. MNP had no overall impact on any of the growth outcomes measured, nor on the longitudinal diarrhoea burden. However, there was a trend towards a small adverse effect of MNP on growth (LAZ and MUAC) among children who were initially non-anaemic, but not among children who were anaemic. MNP had a small beneficial impact on longitudinal diarrhoea prevalence among children without clinically significant IHbD, but a small adverse effect among children with IHbD.

Although there was a significant effect of MNP on concentrations of Hb and Fe status indicators, and a reduction in the prevalence of Fe deficiency, the 8 % reduction in anaemia prevalence at end line was only marginally significant. This lack of a significant impact differs from that found in two meta-analyses, where MNP reduced anaemia by 34 %^(3,4)^, and we have previously discussed the potential reasons for the present results elsewhere^(16)^. The MNP in the present study provided additional micronutrients along with Fe, including folic acid, vitamin B12 and vitamin A; so inadequacy of these nutrients should not have limited the Hb response to the MNP. It is possible that other non-nutritional factors such as IHbD, intestinal helminths and other infections may have contributed to the anaemia burden in the study population. Indeed, we found highly significant differences in baseline anaemia prevalence between the IHbD types as categorised in the present study. Thus, although IHbD did not modify the Hb response to MNP in the present analyses, IHbD was associated with an elevated prevalence of anaemia.

The aforementioned meta-analyses also found an effect of MNP on the prevalence of Fe deficiency^(3)^ and Fe-deficiency anaemia^(4)^; however, the meta-analysis by Salam *et al.*^(4)^ found no impact of MNP on final serum ferritin concentrations, and de Regil *et al.*^(3)^ were unable to determine an impact on ferritin concentrations due to the low quality of evidence in available studies. We found that the provision of MNP containing 6 mg Fe not only decreased the prevalence of low ferritin and high sTfR concentrations (indicative of Fe deficiency) but also increased the mean concentrations of ferritin and reduced mean concentrations of sTfR. As expected, we found that these effects of MNP on Hb, ferritin and prevalence of anaemia and Fe deficiency were greater among children who had low baseline Hb, low ferritin or high sTfR concentrations.

Although we did not detect a modifying effect of IHbD on the impact of MNP on final anaemia prevalence, the presence/ absence of IHbD marginally modified the intervention effect on the prevalence of low ferritin and high sTfR at end line. MNP reduced the prevalence of low ferritin and high sTfR in both children without clinically significant IHbD and those with IHbD, when all IHbD were combined, but it appeared to have a stronger effect among children without IHbD. This was expected, because both ferritin and sTfR are affected not only by Fe deficiency but also by changes in Fe metabolism and erythropoiesis observed in individuals with IHbD^(33)^. Children with IHbD tended to have higher ferritin concentrations at baseline and end line, which is likely due to an increased Fe absorption observed in some IHbD types^(13,15)^. Similarly, in individuals with IHbD, sTfR concentrations are frequently elevated due to increased erythropoiesis^(34)^. Thus, both ferritin and sTfR are affected by IHbD and, therefore, do not reflect Fe status alone. This is in agreement with previous findings that different types of IHbD respond differently to Fe supplementation^(35)^, and it has been shown that hepcidin concentrations and Fe absorption differ by IHbD type^(13,14)^.

In the present study, we found a modifying effect of initial Hb status on final LAZ, MUAC and MUACZ in response to MNP. There was a positive impact on final mean LAZ and final mean MUAC and MUACZ among initially anaemic children, while MNP was associated with a small adverse effect on linear growth among non-anaemic children. Although MNP contained several micronutrients, which could potentially have contributed to this adverse effect, several previous studies have shown an adverse effect of Fe on growth among non-anaemic or Fereplete children. For example, Fe supplementation resulted in lower weight gain compared with placebo in young children in Indonesia^(36)^, and Swedish infants who received Fe supplements from 4 to 9 months of age gained significantly less in length and head circumference than those given placebo^(37)^. This latter study also included a cohort of children in Honduras, where this same effect of reduced length gain was found only in children who were initially non-anaemic, which is similar to the present results. The overall impact of supplementary Fe on young children’s growth has been inconsistent^(38)^, and the underlying mechanisms of reduced growth observed with Fe supplementation in some studies are unknown^(39)^, but supplementary Fe may be more likely to result in adverse effects on growth among non-anaemic or Fe-replete children^(37,40,41)^.

Among the growth outcomes, only final wasting prevalence was modified by IHbD. When investigating this further, MNP seemed to slightly increase the final wasting prevalence only among children without clinically significant IHbD and children with *β*-thalassaemia trait or homozygous Hb E, though it did not reach significance in the latter group. It is known that clinically severe forms of thalassaemia can result in growth restriction^(42,43)^. Similarly, in the present study, we found significant differences in baseline length and LAZ, with the smallest LAZ among children with thalassaemia disease. However, it is not clear why MNP would adversely affect wasting among some subgroups of children without clinically significant IHbD and children with *β*-thalassaemia trait or homozygous Hb E, considering that there was no overall effect of MNP on the final length and final weight; and neither one of these anthropometric outcomes was modified by IHbD at end line. However, in a recent Zn dose–response trial of small quantity lipid-based nutrient supplements (SQ-LNS) among young Burkinabe children, final wasting prevalence was also significantly higher in the group that received SQ-LNS containing 10 mg Zn for 9 months compared with the children who received SQ-LNS without Zn (13.2 *v.* 7.2 %)^(44)^. This negative impact of MNP and SQ-LNS containing 10 mg Zn is puzzling, considering that a meta-analysis of twenty-two studies of preventive Zn supplementation provided as either tablets or syrup, including thirteen studies that provided 10 mg supplemental Zn, found a small, marginally significant, positive effect of Zn on change in weight-for-height z score^(45)^. Moreover, in the present study we found a significantly lower wasting prevalence at end line in the group receiving Zn supplements alone (7 mg Zn/d) compared with the therapeutic Zn supplementation group (20 mg Zn for 10 d during diarrhoea episodes)^(16)^. Thus, it is possible that some other component of the MPN and SQ-LNS may block the effect of Zn on wasting prevalence. Further research is needed to understand these effects.

Overall, MNP had no impact on the longitudinal diarrhoea prevalence or diarrhoea incidence rates in the present study. Thus, providing an MNP with a higher dose of Zn did not result in the beneficial impact on diarrhoea observed in randomised control trials of preventive Zn supplementation provided as tablets or syrups^(45–^^47)^. This may be due to the Fe contained in MNP. In a meta-analysis, Mayo-Wilson et al.^(48)^ found a significant beneficial impact of preventive Zn on diarrhoea incidence among children who received Zn without Fe (risk ratio 0.82 (95 % CI 0.85, 0.89; twenty-two studies)), but this effect was not found among studies where Fe was given along with Zn (risk ratio 1.00 (95 % CI 0.96,1.05); ten studies). While we found no benefit of MNP on diarrhoea, we also found no overall adverse effect of MNP on diarrhoea. This is in contrast to a cluster randomised trial among 6- to 18-month-old children in Pakistan, which found that MNP significantly increased the longitudinal diarrhoea prevalence and incidence of bloody diarrhoea^(49)^. Several other studies also found no difference in diarrhoea or overall morbidity among children who received MNP compared with a control group^(50–^^53)^. However, we found that MNP appeared to have a small beneficial impact on longitudinal diarrhoea prevalence among children without clinically significant IHbD and a small adverse effect among children with IHbD. The magnitude of the adverse effect on diarrhoea was small and the modifying effect was only marginally significant. Nevertheless, public health programmes considering MNP population-based distribution should also strengthen the diarrhoea treatment programme. The mechanism by which MNP might increase diarrhoea among children with IHbD is not clear. MNP has been shown to adversely affect the microbiome in the gut by increasing the abundance of pathogens and causing intestinal inflammation^(54,55)^, but it is not known whether this effect would differ by IHbD type.

To our knowledge, this is the first MNP study that investigated the modifying effects of baseline Fe status and IHbD on various health outcomes. Strengths of the present study include the randomised double-blind study design, large sample size, frequency of follow-up, high participation rates and regular training and rigorous supervision of the data collectors. A limitation of the study is that we were not able to complete assessments of the full set of IHbD in all children. Although we assessed *α*^0^-thalassaemia genes in all children, other genetic tests were performed primarily among children with a suspicion of clinically significant IHbD. In particular, molecular screening for *β*-thalassaemia mutations was carried out in children with either Hb A_2_ > 3.5 % or Hb EF phenotype, and homozygosity of Hb E was tested in all cases with Hb EE as well as Hb EF phenotypes. Thus, although complete diagnosis of all types and variations of IHbD was not possible, the analytic approach allowed for the detection of all clinically significant cases of IHbD.

In summary, low-Fe, high-Zn MNP had a beneficial impact on Hb, ferritin and sTfR concentrations, especially among children with low initial Hb concentration and Fe status. The MNP had no overall impact on growth and longitudinal diarrhoea prevalence, but provision of MNP was associated with a small adverse effect on linear growth among non-anaemic children and on diarrhoea among children with IHbD. The magnitude of observed adverse effects was small but highlighted subgroups who may respond adversely to supplemental MNP. The potential risks and benefits of population-based distribution of MNP must be considered in individual contexts, depending on the prevalence of anaemia and IHbD.

## Supplementary material

For supplementary material/s referred to in this article, please visit https://doi.org/10.1017/S0007114519001715

## References

[1] Black RE, Victora CG, Walker SP, et al. (2013) Maternal and child undernutrition and overweight in low-income and middle-income countries. *Lancet* 382, 427–451. 23746772 10.1016/S0140-6736(13)60937-X

[2] World Health Organization (2016) *WHO Guideline: Multiple Micronutrients Powders for Point-of-use Fortification of Foods Consumed by Children 6-23 Months of Age and Children Aged 2-12 Years.* Geneva, Switzerland: World Health Organization.

[3] De-Regil LM, Jefferds MED & Pena-Rosas JP (2017) Point-of-use fortification of foods with micronutrient powders containing iron in children of preschool and school-age. *Cochrane Database Syst Rev,* issue 11, CD009666. 29168569 10.1002/14651858.CD009666.pub2 PMC6486284

[4] Salam RA, Macphail C, Das JK, et al. (2013) Effectiveness of Micronutrient Powders (MNP) in women and children. *BMC Public Health* 13, Suppl. 3, S22. 24564207 10.1186/1471-2458-13-S3-S22 PMC3847468

[5] Pasricha SR & Drakesmith H (2016) Iron deficiency anemia: problems in diagnosis and prevention at the population level. *Hematol Oncol Clin North Am* 30, 309–325. 27040956 10.1016/j.hoc.2015.11.003

[6] Home Fortification Technical Advisory Group (2018) Home fortification activities worldwide. http://www.hftag.org (accessed February 2018).

[7] Rivella S & Giardina JP (2013) Thalassemia syndromes. In *Hematology: Basic Principles and Practice,* pp. 505–535 [Hoffman R, Benz EJ, Silberstein L, Heslop H, Weitz J and Anastasi J, editors]. Philadelphia, PA: Elsevier.

[8] Vichinsky E (2007) Hemoglobin E syndromes. *Hematology Am Soc Hematol Educ Program* 2007, 79–83. 10.1182/asheducation-2007.1.79 18024613

[9] Modell B & Darlison M (2008) Global epidemiology of haemoglobin disorders and derived service indicators. *Bull World Health Organ* 86, 480–487. 18568278 10.2471/BLT.06.036673 PMC2647473

[10] Weatherall DJ & Clegg JB (2001) Inherited haemoglobin disorders: an increasing global health problem. *Bull World Health Organ* 79, 704–712. 11545326 PMC2566499

[11] Fucharoen S & Winichagoon P (2000) Clinical and hematologic aspects of hemoglobin E beta-thalassemia. *Curr Opin Hematol* 7, 106–112. 10698297 10.1097/00062752-200003000-00006

[12] George J, Yiannakis M, Main B, et al. (2012) Genetic hemoglobin disorders, infection, and deficiencies of iron and vitamin A determine anemia in young Cambodian children. *J Nutr* 142, 781–787. 22378325 10.3945/jn.111.148189 PMC3301994

[13] Zimmermann MB, Fucharoen S, Winichagoon P, et al. (2008) Iron metabolism in heterozygotes for hemoglobin E (HbE), alpha-thalassemia 1, or beta-thalassemia and in compound heterozygotes for HbE/beta-thalassemia. *Am J Clin Nutr* 88, 1026–1031. 18842790 10.1093/ajcn/88.4.1026

[14] Jones E, Pasricha SR, Allen A, et al. (2015) Hepcidin is suppressed by erythropoiesis in hemoglobin E beta-thalassemia and beta-thalassemia trait. *Blood* 125, 873–880. 25519750 10.1182/blood-2014-10-606491 PMC4321326

[15] Guimaraes JS, Cominal JG, Silva-Pinto AC, et al. (2015) Altered erythropoiesis and iron metabolism in carriers of thalassemia. *Eur J Haematol* 94, 511–518. 25307880 10.1111/ejh.12464 PMC4393762

[16] Barffour M, Hinnouho G, Kounnavong S, et al. (2019) Effects of daily zinc, daily multiple micronutrient powder, or therapeutic zinc supplementation for diarrhea prevention on physical growth, anemia, and micronutrient status in rural Laotian children: a randomized controlled trial. *J Pediatr* 207, 80–89.e2. 30580974 10.1016/j.jpeds.2018.11.022 PMC6448681

[17] Barffour MA, Hinnouho GM, Kounnavong S, et al. (2018) Effects of two forms of daily preventive zinc and therapeutic zinc supplementation for diarrhea on diarrhea and acute respiratory tract infections in Laotian children. *Curr Dev Nutr* 2, OR32–05.

[18] Wessells KR, Brown KH, Kounnavong S, etal. (2018) Comparison of two forms of daily preventive zinc supplementation versus therapeutic zinc supplementation for diarrhea on young children’s physical growth and risk of infection: study design and rationale for a randomized controlled trial. *BMC Nutr* 4, 39. 32153900 10.1186/s40795-018-0247-6 PMC7050875

[19] WHO Multicentre Growth Reference Study Group (2006) *WHO Child Growth Standards: Length/height-for-age, Weight-for-age, Weight-for-length, Weight-for-height and Body Mass Index-for-age: Methods and Development.* Geneva: WHO.

[20] European Union, Ministry of Health, MMG, UNICEF (2011) *What is SuperKid? Counseling Cards. Ministry of Health*. Vientiane: Lao PDR.

[21] Coates J, Swindale A & Bilinsky P (2007) Household Food Insecurity Access Scale (HFIAS) for Measurement of Food Access: Indicator Guide. Washington, DC: USAID.

[22] de Onis M, Onyango AW, Van den Broeck J, et al. (2004) Measurement and standardization protocols for anthropometry used in the construction of a new international growth reference. *Food Nutr Bull* 25, S27–S36. 15069917 10.1177/15648265040251S104

[23] Hess SY, Hinnouho GM, Barffour MA, et al. (2018) First field test of an innovative, wider tape to measure mid-upper arm circumference in young Laotian children. *Food Nutr Bull* 39, 28–38. 29258337 10.1177/0379572117742502

[24] World Health Organization (2008) *Indicators for Assessing Infant and Young Child Feeding Practices. Part I: Definition.* Geneva: WHO.

[25] World Health Organization (2010) *Indicators for Assessing Infant and Young Child Feeding Practices. Part II: Measurement.* Geneva: WHO.

[26] Erhardt JG, Estes JE, Pfeiffer CM, et al. (2004) Combined measurement of ferritin, soluble transferrin receptor, retinol binding protein, and C-reactive protein by an inexpensive, sensitive, and simple sandwich enzyme-linked immunosorbent assay technique. *J Nutr* 134, 3127–3132. 15514286 10.1093/jn/134.11.3127

[27] Yamsri S, Sanchaisuriya K, Fucharoen G, et al. (2010) Prevention of severe thalassemia in northeast Thailand: 16 years of experience at a single university center. *Prenat Diagn* 30, 540–546. 20509153 10.1002/pd.2514

[28] Sae-ung N, Fucharoen G, Sanchaisuriya K, et al. (2007) Alpha(0)-thalassemia and related disorders in northeast Thailand: a molecular and hematological characterization. *Acta Haematol* 117, 78–82. 17106191 10.1159/000096857

[29] WHO Multicentre Growth Reference Study Group (2007) *WHO Child Growth Standards: Head Circumference-for-age, Arm circumference-for-age, Triceps Skinfold-for-age and Subscapular Skinfold-for-age: Methods and Development.* Geneva: WHO.

[30] Hess SY, Barffour MA & Hinnouho GM (2018) Lao Zinc Study. Open Science Framework. https://osf.io/5bq9c (accessed December 2018).

[31] Namaste SM, Rohner F, Huang J, et al. (2017) Adjusting ferritin concentrations for inflammation: Biomarkers Reflecting Inflammation and Nutritional Determinants of Anemia (BRINDA) project. *Am J Clin Nutr* 106, Suppl. 1, 359S–371S. 28615259 10.3945/ajcn.116.141762 PMC5490647

[32] Rohner F, Namaste SM, Larson LM, et al. (2017) Adjusting soluble transferrin receptor concentrations for inflammation: Biomarkers Reflecting Inflammation and Nutritional Determinants of Anemia (BRINDA) project. *Am J Clin Nutr* 106, Suppl. 1, 372S–382S. 28615256 10.3945/ajcn.116.142232 PMC5490651

[33] Lynch S, Pfeiffer CM, Georgieff MK, et al. (2018) Biomarkers of Nutrition for Development (BOND)-iron review. *J Nutr* 148, 1001S–1067S. 29878148 10.1093/jn/nxx036 PMC6297556

[34] World Health Organization (2014) *Serum Transferrin Receptor Levels for the Assessment of Iron Status and Iron Deficiency in Populations. Vitamin and Mineral Nutrition Information System.* Geneva: WHO.

[35] Sanchaisuriya K, Fucharoen S, Ratanasiri T, et al. (2007) Effect of the maternal betaE-globin gene on hematologic responses to iron supplementation during pregnancy. *Am J Clin Nutr* 85, 474–479. 17284746 10.1093/ajcn/85.2.474

[36] Idjradinata P, Watkins WE & Pollitt E (1994) Adverse effect of iron supplementation on weight gain of iron-replete young children. *Lancet* 343, 1252–1254. 7910275 10.1016/s0140-6736(94)92151-2

[37] Dewey KG, Domellöf M, Cohen RJ, et al. (2002) Iron supplementation affects growth and morbidity of breast-fed infants: results of a randomized trial in Sweden and Honduras. *J Nutr* 132, 3249–3255. 12421836 10.1093/jn/132.11.3249

[38] Pasricha SR, Hayes E, Kalumba K, et al. (2013) Effect of daily iron supplementation on health in children aged 4–23 months: a systematic review and meta-analysis of randomised controlled trials. *Lancet Glob Health* 1, e77–e86. 25104162 10.1016/S2214-109X(13)70046-9

[39] Lonnerdal B (2017) Excess iron intake as a factor in growth, infections, and development of infants and young children. *Am J Clin Nutr* 106, 1681S–1687S. 29070544 10.3945/ajcn.117.156042 PMC5701711

[40] Majumdar I, Paul P, Talib VH, et al. (2003) The effect of iron therapy on the growth of iron-replete and iron-deplete children. *J Trop Pediatr* 49, 84–88. 12729289 10.1093/tropej/49.2.84

[41] Lind T, Seswandhana R, Persson LA, et al. (2008) Iron supplementation of iron-replete Indonesian infants is associated with reduced weight-for-age. *Acta Paediatr* 97, 770–775. 18422809 10.1111/j.1651-2227.2008.00773.x

[42] Vogiatzi MG, Macklin EA, Trachtenberg FL, et al. (2009) Differences in the prevalence of growth, endocrine and vitamin D abnormalities among the various thalassaemia syndromes in North America. *Br J Haematol* 146, 546–556. 19604241 10.1111/j.1365-2141.2009.07793.x PMC2798591

[43] Soliman AT, De Sanctis V, Yassin M, et al. (2017) Growth and growth hormone – insulin like growth factor -I (GH-IGF-I) axis in chronic anemias. *Acta Biomed* 88, 101–111. 28467344 10.23750/abm.v88i1.5744 PMC6166184

[44] Hess SY, Abbeddou S, Jimenez EY, et al. (2015) Small-quantity lipid-based nutrient supplements, regardless of their zinc content, increase growth and reduce the prevalence of stunting and wasting in young Burkinabe children: a cluster-randomized trial. *PLOS ONE* 10, e0122242. 25816354 10.1371/journal.pone.0122242 PMC4376671

[45] Brown KH, Peerson JM, Baker SK, et al. (2009) Preventive zinc supplementation among infants, preschoolers, and older prepubertal children. *Food Nutr Bull* 30, S12–S40. 19472600 10.1177/15648265090301S103

[46] Mayo-Wilson E, Junior JA, Imdad A, et al. (2014) Zinc supplementation for preventing mortality, morbidity, and growth failure in children aged 6 months to 12 years of age. *Cochrane Database Syst Rev,* issue 5, CD009384. 24826920 10.1002/14651858.CD009384.pub2

[47] Yakoob MY, Theodoratou E, Jabeen A, et al. (2011) Preventive zinc supplementation in developing countries: impact on mortality and morbidity due to diarrhea, pneumonia and malaria. *BMC Public Health* 11, Suppl. 3, S23. 21501441 10.1186/1471-2458-11-S3-S23 PMC3231897

[48] Mayo-Wilson E, Imdad A, Junior J, et al. (2014) Preventive zinc supplementation for children, and the effect of additional iron: a systematic review and meta-analysis. *BMJ Open* 4, e004647. 10.1136/bmjopen-2013-004647 PMC4067863 24948745

[49] Soofi S, Cousens S, Iqbal SP, et al. (2013) Effect of provision of daily zinc and iron with several micronutrients on growth and morbidity among young children in Pakistan: a cluster-randomised trial. *Lancet* 382, 29–40. 23602230 10.1016/S0140-6736(13)60437-7

[50] Sampaio DL, Mattos AP, Ribeiro TC, et al. (2013) Zinc and other micronutrients supplementation through the use of sprinkles: impact on the occurrence of diarrhea and respiratory infections in institutionalized children. *J Pediatr (Rio J*) 89, 286–293. 23664200 10.1016/j.jped.2012.11.004

[51] Osei AK, Rosenberg IH, Houser RF, et al. (2010) Community-level micronutrient fortification of school lunch meals improved vitamin A, folate, and iron status of school children in Himalayan villages of India. *J Nutr* 140, 1146–1154. 20410083 10.3945/jn.109.114751

[52] Inayati DA, Scherbaum V, Purwestri RC, et al. (2012) Combined intensive nutrition education and micronutrient powder supplementation improved nutritional status of mildly wasted children on Nias Island, Indonesia. *Asia Pac J Clin Nutr* 21, 361–373. 22705425

[53] Giovannini M, Sala D, Usuelli M, et al. (2006) Double-blind, placebo-controlled trial comparing effects of supplementation with two different combinations of micronutrients delivered as sprinkles on growth, anemia, and iron deficiency in Cambodian infants. *J Pediatr Gastroenterol Nutr* 42, 306–312. 16540800 10.1097/01.mpg.0000189363.07040.4b

[54] Jaeggi T, Kortman GA, Moretti D, et al. (2014) Iron fortification adversely affects the gut microbiome, increases pathogen abundance and induces intestinal inflammation in Kenyan infants. *Gut*. 10.1136/gutjnl-2014-307720 25143342

[55] Paganini D & Zimmermann MB (2017) The effects of iron fortification and supplementation on the gut microbiome and diarrhea in infants and children: a review. *Am J Clin Nutr* 106, 1688S–1693S. 29070552 10.3945/ajcn.117.156067 PMC5701709

