# Supplementary material for: Iron status and inherited haemoglobin disorders modify the effects of micronutrient powders on linear growth and morbidity among young Lao children in a double-blind randomised trial
Source: Br J Nutr. 2019 Mar 8;122(8):895–909. doi: 10.1017/S0007114519001715 (PMC7672373; doi:10.1017/S0007114519001715)
Supplement: Supplementary file 1 [file BJN-122-08-895-s001.docx]

Supplementary Table S1: Baseline characteristics of young children, mothers and households among those with endline anthropometry outcomes*

|  | MNP | Placebo | p-value |
| --- | --- | --- | --- |
| N | 701 | 740 |  |
| Children |  |  |  |
| Enrollment age (months) | 14.3 ± 5.0 | 14.1 ± 5.1 | 0.426 |
| Male, % | 50.7 | 52.0 | 0.618 |
| Adequate dietary diversity at baseline^†^, % | 13.6 | 15.8 | 0.273 |
| Minimum meal frequency at baseline^†^, % | 60.7 | 57.9 | 0.328 |
| Consumption of iron rich foods at baseline^†^, % | 67.6 | 64.8 | 0.297 |
| Proportion of days with adequate dietary diversity^‡^ | 0.30 ± 0.28 | 0.30 ± 0.28 | 0.845 |
| Proportion of days with minimum meal frequency^‡^ | 0.61 ± 0.36 | 0.62 ± 0.35 | 0.533 |
| Proportion of days with iron rich foods^‡,§^ | 0.85 ± 0.19 | 0.85 ± 0.19 | 0.540 |
| Mothers |  |  |  |
| Age (years) | 27.1 ± 6.0 | 27.0 ± 6.1 | 0.759 |
| Maternal height (cm) | 150.4 ± 5.1 | 150.7 ± 5.4 | 0.300 |
| Maternal BMI (kg/m^2^) | 21.5 ± 2.7 | 21.4 ± 3.0 | 0.512 |
| Years of education | 4.7 ± 3.5 | 4.6 ± 3.5 | 0.766 |
| Buddhist, % | 87.1 | 90.1 | 0.161 |
| Married, % | 97.3 | 95.3 | 0.068 |
| Households |  |  |  |
| Food secure^ǁ^, % | 32.6 | 34.2 | 0.829 |
| Number of children under 5y | 1.4 ± 0.6 | 1.5 ± 0.7 | 0.446 |
| Improved walls and floor, % | 31.6 | 32.1 | 0.837 |
| Number of rooms | 2.5 ± 1.0 | 2.5 ± 1.0 | 0.877 |
| Number of poultry owned | 9.3 ± 11.1 | 9.4 ± 10.9 | 0.918 |

* Values are Mean ± SD or %.

^†^ Baseline infant and young child feeding (IYCF) practices during 24 hr prior to enrollment^(24-25)^.

^‡^ IYCF practices assessed every four weeks throughout the study^(24-25)^; proportion of observed days with the positive feeding behavior during study.

^§^ Iron rich foods did not include the MNP provided by the study.

^ǁ^ Food security assessed using the Household Food Insecurity Access Scale^(21)^.

Supplementary Table S2: Effect of a low iron, high zinc MNP on hemoglobin and iron status among young children in the Lao Zinc Study*

|  | MNP | | Placebo | | Minimally adjusted^†^ comparison at endline (95% CI) | Fully adjusted^‡^ comparison at endline (95% CI) |
| --- | --- | --- | --- | --- | --- | --- |
|  | Baseline | Endline | Baseline | Endline |  |  |
| **Hemoglobin, N** | 686 | | 720 | |  |  |
| Hemoglobin (g/L) | 107.4 ± 10.6 | 111.4 ± 10.7 | 108.0 ± 10.1 | 110.6 ± 10.9 | 1.07 (0.05, 2.09)* | 1.20 (0.17, 2.22)* |
| Anemia (Hb < 110g/L), % | 391 (57.0) | 272 (39.7) | 388 (53.9) | 311 (43.2) | 0.90 (0.80, 1.01) | 0.89 (0.80, 1.00) |
| **Iron status**^§^**, N** | 513 | | 522 | |  |  |
| Ferritin (µg/L) | 16.9 (8.6, 29.5) | 25.0 (14.5, 39.4) | 17.2 (9.8, 30.4) | 19.0 (10.5, 30.9) | 1.30 (1.21, 1.40)* | 1.30 (1.20, 1.40)* |
| Low ferritin (pF*<*12 µg/L), % | 178 (34.7) | 104 (20.3) | 172 (33.0) | 160 (30.7%) | 0.65 (0.54, 0.79)* | 0.64 (0.53, 0.78)* |
| sTfR (mg/L) | 9.07 (7.61, 11.60) | 8.01 (6.77, 9.84) | 8.94 (7.36, 10.99) | 8.44 (7.21, 10.48) | 0.93 (0.90, 0.95)* | 0.93 (0.90, 0.95)* |
| High sTfR (sTfR*>*8.3 mg/L), % | 315 (61.4) | 232 (45.2) | 304 (58.2) | 278 (53.3) | 0.86 (0.77, 0.96)* | 0.85 (0.76, 0.95)* |
| Iron deficiency^ǁ^, % | 343 (66.9) | 261 (50.9) | 339 (64.9) | 304 (58.2) | 0.87 (0.79, 0.96)* | 0.85 (0.77, 0.95)* |
| Iron deficiency^ǁ^ anemia, % | 220 (43.9) | 136 (27.1) | 204 (39.5) | 160 (31.0) | 0.87 (0.73, 1.03) | 0.85 (0.72, 1.02) |

Hb, hemoglobin; MNP, micronutrient powder; pF, plasma ferritin; sTfR, soluble transferrin receptor

* Values are mean ± SD with difference in means for comparison, or n (%) and prevalence ratio for comparison, or median (Q1, Q3) and geometric mean ratio for comparison.

^†^ Minimally adjusted for baseline value, age at enrolment, and district.

^‡^ Adjusted for baseline value, age at enrolment and district, and potentially adjusted (where P for correlation<0.1) for maternal age at baseline, height, BMI, and education, child sex, month of enrolment, adequate dietary diversity at baseline, minimum meal frequency at baseline, iron rich foods consumed at baseline, number of children under 5 in the household, household food insecurity score, socio-economic index, average adequate dietary diversity, average consumption of iron rich foods, and baseline growth status.

^§^ Iron status indicators adjusted for inflammation. Continuous outcomes log transformed for analysis.

^ǁ^ Iron deficiency defined as ferritin < 12 µg/L and/or sTfR > 8.3 mg/L

Statistically significant *P < 0.05

Supplementary Table S3: Effect of a low iron, high zinc MNP on growth of young children in the Lao Zinc Study*

|  | MNP | | Placebo | | Minimally adjusted^†^  comparison (95% CI) | Fully adjusted^‡^  comparison (95% CI) |
| --- | --- | --- | --- | --- | --- | --- |
|  | Baseline | Endline | Baseline | Endline |  |  |
| N | 701 | | 740 | |  |  |
| Length (cm) | 72.5 ± 5.4 | 79.2 ± 4.8 | 72.5 ± 5.6 | 79.3 ± 4.9 | -0.08 (-0.21, 0.06) | -0.06 (-0.19, 0.07) |
| LAZ | -1.76 ± 1.07 | -1.95 ± 0.99 | -1.67 ± 1.09 | -1.87 ± 1.00 | -0.01 (-0.06, 0.03) | -0.01 (-0.05, 0.03) |
| Stunting, % (LAZ < -2 SD) | 287 (41.0) | 336 (48.0) | 276 (37.3) | 317 (42.8) | 1.05 (0.95, 1.16) | 1.04 (0.95, 1.15) |
| Weight (kg) | 8.28 ± 1.30 | 9.58 ± 1.32 | 8.33 ± 1.31 | 9.64 ± 1.33 | -0.01 (-0.05, 0.04) | -0.01 (-0.05, 0.04) |
| WAZ | -1.46 ± 1.02 | -1.54 ± 0.93 | -1.37 ± 0.99 | -1.47 ± 0.91 | 0.00 (-0.04, 0.04) | 0.00 (-0.04, 0.04) |
| Underweight, % (WAZ < -2 SD) | 212 (30.2) | 197 (28.1) | 176 (23.8) | 203 (27.4) | 0.90 (0.78, 1.04) | 0.91 (0.78, 1.05) |
| WLZ | -0.73 ± 0.96 | -0.74 ± 0.85 | -0.67 ± 0.94 | -0.71 ± 0.82 | 0.02 (-0.04, 0.07) | 0.00 (-0.05, 0.06) |
| Wasting, % (WLZ < -2 SD) | 58 (8.3) | 39 (5.6) | 55 (7.4) | 35 (4.7) | 1.22 (0.80, 1.86) | 1.36 (0.89, 2.08) |
| MUAC (cm) | 13.78 ± 1.00 | 13.97 ± 0.96 | 13.82 ± 1.00 | 14.01 ± 0.94 | -0.01 (-0.07, 0.04) | -0.03 (-0.09, 0.03) |
| MUACZ | -0.69 ± 0.90 | -0.85 ± 0.83 | -0.65 ± 0.88 | -0.81 ± 0.80 | -0.01 (-0.06, 0.04) | -0.02 (-0.08, 0.03) |
| Low MUACZ, % (MUACZ*<*-2 SD) | 48 (6.8) | 58 (8.3) | 45 (6.1) | 49 (6.6) | 1.14 (0.81, 1.60) | 1.14 (0.81, 1.60) |

LAZ, length-for-age z-score; MNP, micronutrient powder; MUAC, mid-upper arm circumference; MUACZ, mid-upper arm circumference z-score; WAZ, weight-for-age z-score; WLZ, weight-for-length z-score

*Values are mean ± SD with difference in means for comparison or n (%) and prevalence ratio for comparison.

^†^Minimally adjusted for baseline value, age at enrolment, and district.

^‡^Adjusted for baseline value, age at enrolment and district, and potentially adjusted (where p for correlation<0.1) for maternal age at baseline, height, BMI, and education, child sex, month of enrolment, adequate dietary diversity at baseline, minimum meal frequency at baseline, iron rich foods consumed at baseline, number of children under 5 in the household, household food insecurity score, socio-economic index, average adequate dietary diversity, average consumption of iron rich foods, and anthropometris
